# Nucleation and growth of (10^¯^11) semi-polar AlN on (0001) AlN by Hydride Vapor Phase Epitaxy

**DOI:** 10.1038/srep26040

**Published:** 2016-05-17

**Authors:** Ting Liu, Jicai Zhang, Xujun Su, Jun Huang, Jianfeng Wang, Ke Xu

**Affiliations:** 1 Platform for Characterization and Test, Suzhou Institute of Nano-tech and Nano-bionics, CAS, Suzhou, 215123, China; 2 Suzhou Nanowin Science and Technology Co., Lt/d, Suzhou, 215123, China

## Abstract

Wurtzite AlN is widely used for deep ultraviolet optoelectronic devices (DUV), which are generally grown along the [0001]-direction of the wurtzite structure on currently available substrates. However, huge internal electrostatic fields are presented within the material along [0001] axis induced by piezoelectric and spontaneous polarization, which has limited the internal quantum efficiency of AlN based DUV LEDs dramatically. The internal fields can be strongly reduced by changing the epitaxial growth direction from the conventional polar *c*-*direction* into less polar crystal directions. Twinned crystal is a crystal consisting of two or more domains with the same crystal lattice and composition but different crystal orientations. In other words, twins can be induced to change crystal directions. In this work we demonstrated that the epitaxial growth of (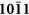) semi-polar AlN on (0001) AlN by constructing (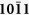) and (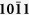) twin structures. This new method is relative feasible than conventional methods and it has huge prospect to develop high-quality semi-polar AlN.

As an important member of the group-III nitrides, aluminum nitride (AlN) has direct bandgap of 6.2 eV with outstanding material properties. It is a promising material for deep ultraviolet optoelectronic devices. These devices such as deep ultraviolet light-emitting diodes (DUV LEDs) are generally grown along the [0001]-direction (usually called *c-direction*) of the wurtzite structure on currently available substrates, for example *c-plane* sapphire. Due to the mismatch-induced biaxial strain and the lattice geometry of group-III nitrides, huge internal electrostatic fields induced by piezoelectric and spontaneous polarization are presented within the material along [0001] axis^1^. This internal field generally results in the band bending effect in quantum wells, which induces some undesirable effects in the quantum wells grown in the direction, such as spatial separation of the wave functions of electrons and holes^2^. As consequence, there is a reduced recombination rate of carriers and a shift in the emission wavelength, which is known as quantum confined Stark effect^3^. AlN based DUV LEDs have been developed for near twenty years^4^. However, the internal quantum efficiency is still far lower than that of their counterparts emitting in blue region. The reasons are complicated. One of them is the high contents of impurities, such as oxygen and carbon. In our case (see Supplementary Fig. S1) both contents are around 10^18^ cm^−3^ which would not reduce the quantum efficiency significantly. Another important reason is the presence of electrostatic fields in the active regions induced by the polarization field along [0001] axis. The internal fields can be strongly reduced by changing the epitaxial growth direction from the conventional polar *c*-*direction* into less polar crystal directions^5^. Based on this idea, researchers have made great efforts to fabricate semi-polar AlN on *r*-sapphire, *r*-LiAlO2, *a*-ZnO, *a*- and *m-plane* SiC^6^,^7^,^8^,^9^,^10^. However, the epitaxy on such non-*c-plane* wafers is much more challenging than that on conventional polar *c-plane* sapphire or SiC^11^. As we know, twinned crystal is a crystal consisting of two or more domains with the same crystal lattice and composition (i.e. the same mineral) but different crystal orientations^12^. In other words, twins can be induced to change crystal directions. In this work we demonstrated that the epitaxial growth of (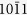) semi-polar AlN on *c-plan*e substrates by constructing (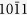) and (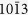) twin structures. This new method is relative feasible than conventional methods. As a result, it has huge prospect to develop high-quality semi-polar AlN.

## Results

**Figure 1:**
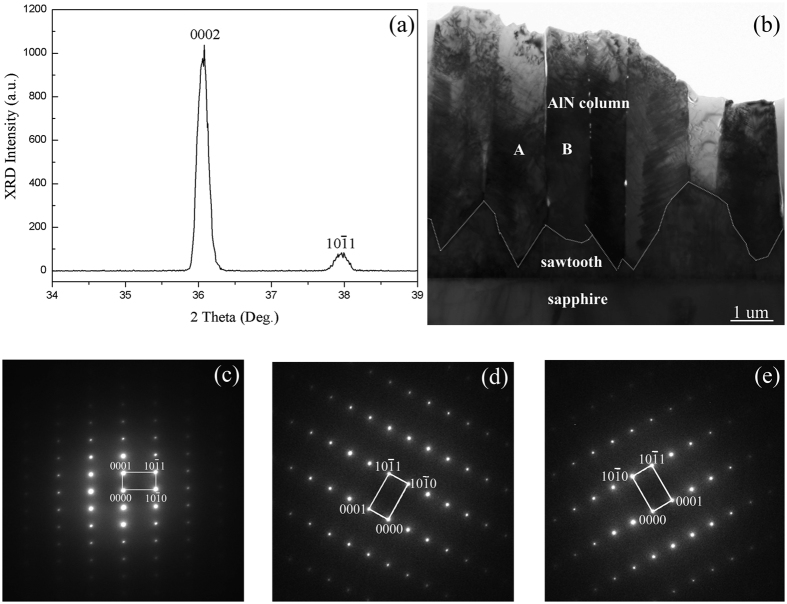
Characterization of AlN film grown on *c-plane* sapphire. (**a**) X-ray 2θ/ω scan of AlN thick film; (**b**) Cross-sectional TEM image of AlN thick film, which contains the sawtooth layer and the columnar layer; (**c**) SAED pattern taken from the sawtooth layer of (**b**); (**d,e**) are the SAED patterns taken from the neighboring column A and B in (**b**), respectively.

Figure 1a shows the X-ray 2θ/ω scan of AlN film grown on *c-plane* sapphire at 1300 °C. Besides the sharp peak of 0002 diffraction at 36.02°, an additional peak is observed around 38° which is identified as 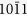 diffraction peak of wurtzite AlN. In order to explore the origin of 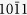 peak, TEM measurement was performed. Figure 1b shows the cross sectional morphology of the AlN thick film. It is clearly observed that this film is composed of two layers with different cross-sectional shapes. The initial layer looks like irregular sawtooth, whose border has been marked by dashed lines. The second layer is the columnar structure grown on the sawtooth layer, where distinct boundaries could be seen between neighboring columns. The selected area electron diffraction (SAED) pattern shown in Fig. 1c proves that the sawtooth layer is along [0001] direction. Figure 1d,e are SAED patterns corresponding to the neighboring columns A and B marked in Fig. 1b. Although the two columns have different distributions of diffraction spots, both of them grow along [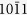] direction instead of the initial [0001] direction. Therefore, 0002 and 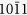 diffraction peaks in Fig. 1a correspond to the AlN sawtooth layer and AlN columnar layer, respectively. According to a preliminary analysis, this AlN film grew along polar direction firstly and then changed to semi-polar direction.

**Figure 2:**
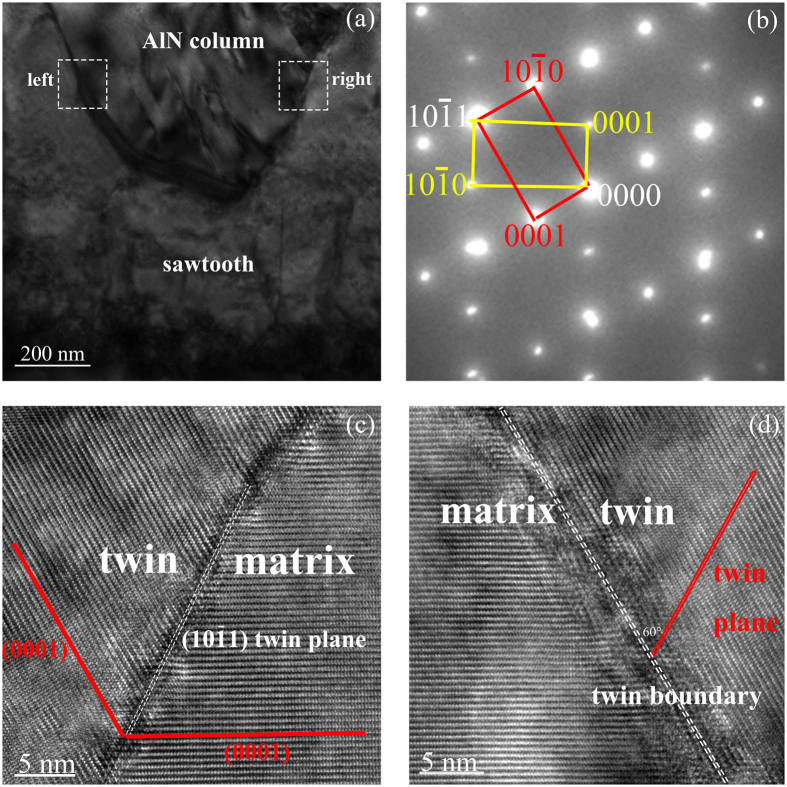
Characterization of 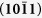 twin boundary with TEM. (**a**) The enlarged image of the interface between the sawtooth and the column. (**b**) SAED pattern corresponding to the left and right interfaces between the sawtooth and the column, which indicates that the two interfaces are (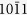) twin boundaries. (**c**) HRTEM image taken from the right interface which indicates it is (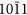) coherent twin boundary. (**d**) HRTEM image taken from the left interface which indicates it is (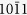) incoherent twin boundary.

In order to understand what happened during the growth, the structural characters of interfaces between the sawtooth layer and the columnar layer were studied. Figure 2a is the enlarged image of interfaces between a column and the sawtooth layer. The two interfaces have the same electron diffraction spots shown in Fig. 2b. It contains two sets of diffraction spots, which have symmetrically geometric distribution. The yellow spots and the red spots reflect the crystallographic information of the sawtooth layer and the column, respectively. The yellow diffraction pattern and the red diffraction pattern are along [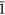
2
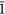
0] and [1
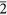
10] zone axis respectively. The common spot shared by the two sets of patterns is identified as the crystal plane (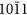). Thus, the two interfaces are proved to be (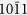) twin boundaries^13^. However, distributions of crystal lattices on the two interfaces are different, which could be observed from the high resolution TEM (HRTEM) images. For the right interface (Fig. 2c), the twin and the matrix distribute symmetrically about the twin boundary (the white dashed line), which indicates that this is a (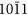) coherent twin boundary. While for the interface on the left (Fig. 2d), the actual twin boundary deviated strikingly from the twinning plane (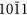) (marked with broken line), with a deviation greater than 50°. Besides, this twin boundary is almost parallel to the basal plane of the columnar layer. This asymmetrical structure is defined as (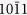) incoherent twin boundary. Actually Zhang *et al.* has observed the similar incoherent twin boundaries in {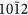} twin system of Co and Mg previously^14^. They attributed the large deviation of the actual twin boundary from the twinning plane (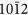) to the atomic shuffling mechanism^15^,^16^. Since shuffling does not have to be confined in the (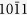) twinning plane, it is permissible for the actual twin boundaries to migrate far off the (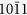) plane during twin growth^17^.

## Discussion

Just by (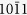) twin growth mode, AlN thick film grown on (0001) sapphire substrate changed growth direction from (0001) polar to (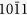) semi-polar. Therefore, the formation of (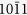) twin boundary is crucial for the change of growth direction. As (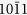) twin structures are formed on the (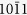) facets, the formation of the first sawtooth layer is the precondition. This process could be explained by the equilibrium crystal shape theory (ECS)^18^,^19^. ECS theory indicates that the surface energies of crystal plane vary with the growth temperature. The surface free energy of a certain crystal plane ω is^20^


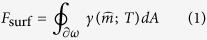


where 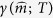 is defined as the surface free energy per unit area, which is the function of the temperature T and the crystal orientation 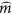
21.


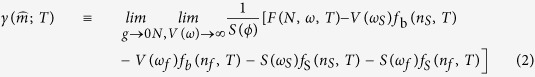


In this formula, *S(Φ)* is the area of *Φ*. A sample of N particles in a rigid container ω has a canonical (Helmholtz) free energy *F* (*N*, *ω*, *T*). *f*_*b*_(*n*_*s*_, *T*) and *f*_*b*_(*n*_*f*_, *T*) are the bulk (Helmholtz) free energies per unit volume of the coexisting uniform phases. The first two subtractions remove the bulk-free energy contributions of the solid-occupied and fluid-occupied portions of the overall region *ω* (*V*(*ω*) = *V*(*ω*_*s*_) + *V*(*ω*_*f*_)); the second two remove the surface-free energy contributions of the solid/wall and fluid/wall boundaries, in which *S*(*ω*) is the area of the surface ∂ω of *ω*. If the surface are very smooth and composed of only one special plane, the surface energy will increase with the increase of temperature (see Supplementary Fig. S2). Actually, the surface will be composed of a serious of micro steps if it shows undulate, which will change the surface free energy of the system.

**Figure 3:**
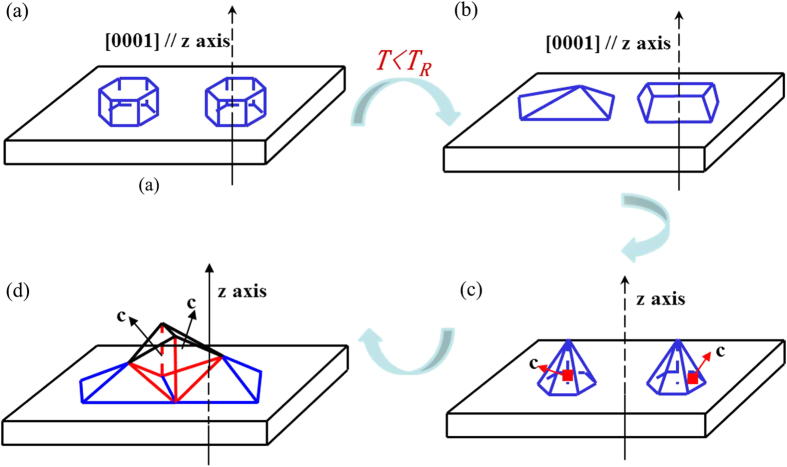
ECS model. (**a**) When the growth temperature is high enough, AlN grows along [0001] direction and (0001) is the facet on surface. (**b**) Other facet on surface instead of (0001) occurs while the growth temperature decreases. (**c**) (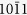) plane becomes facet on surface when the growth temperature is lower than 1390 °C. (**d**) (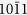) twin boundaries formed at the basis of (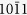) facets on surface.

The evolution process with different temperature is shown in Fig. 3. For AlN grown on (0001) sapphire substrate, if the temperature is high enough, the facet on surface is (0001) plane (as shown in Fig. 3a) since its free energy is the least. The *F*_*surf*_ will change for a certain plane when growth temperature decreases. If the temperature is lower than a certain value, other crystal plane, such as (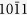) and (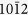), will have lower surface energy than that of (0001). Figure 3b shows that the crystal grains are still [0001]-directed monocrystal but the facets on surface are no longer (0001). The calculation indicates that {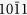} crystal planes become the facets on surface when the growth temperature is less than 1390 °C (Fig. 3c) under the growth conditions used in AlN growth on sapphire of this work. In our case, the actual growth temperature is 1300 °C, which is below the critical value of 1390 °C. Consequently, ECS theory has well clarified the appearance of {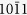} facets on surface in the AlN sawtooth layer.

If AlN continues growing on the previous {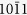} facets of the sawtooth layer, *c-direction* could not keep paralleling the *z-axis* (parallel with [0001]) any longer. Apparently, once the subsequent crystal changes growth orientation, large-angle grain boundaries must be developed at {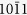} facets on surface. The formation of large-angle grain boundaries means that the energy of the crystal will increase significantly. Contrary to the common boundary, the energy of twin boundary is much lower^22^. Actually, the energy of coherent twin boundary is only one-tenth of that of a common one while the value for an incoherent twin boundary is only a half. Therefore, forming twin boundaries at {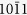} facets on surface, which is shown in Fig. 3d, contributes to remaining a low-energy and more stable state of the crystal. Furthermore, there are possible twin boundaries defined by (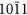), (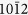) and (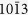) in wurtzite structures^23^,^24^. The appearance of the {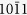} facets on surface makes it possible to develop (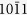) twin boundaries. For the sake of the least energy of the crystal and satisfying certain geometry, the formation of (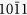) twin boundaries on {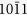} facets on surface are reasonable.

**Figure 4:**
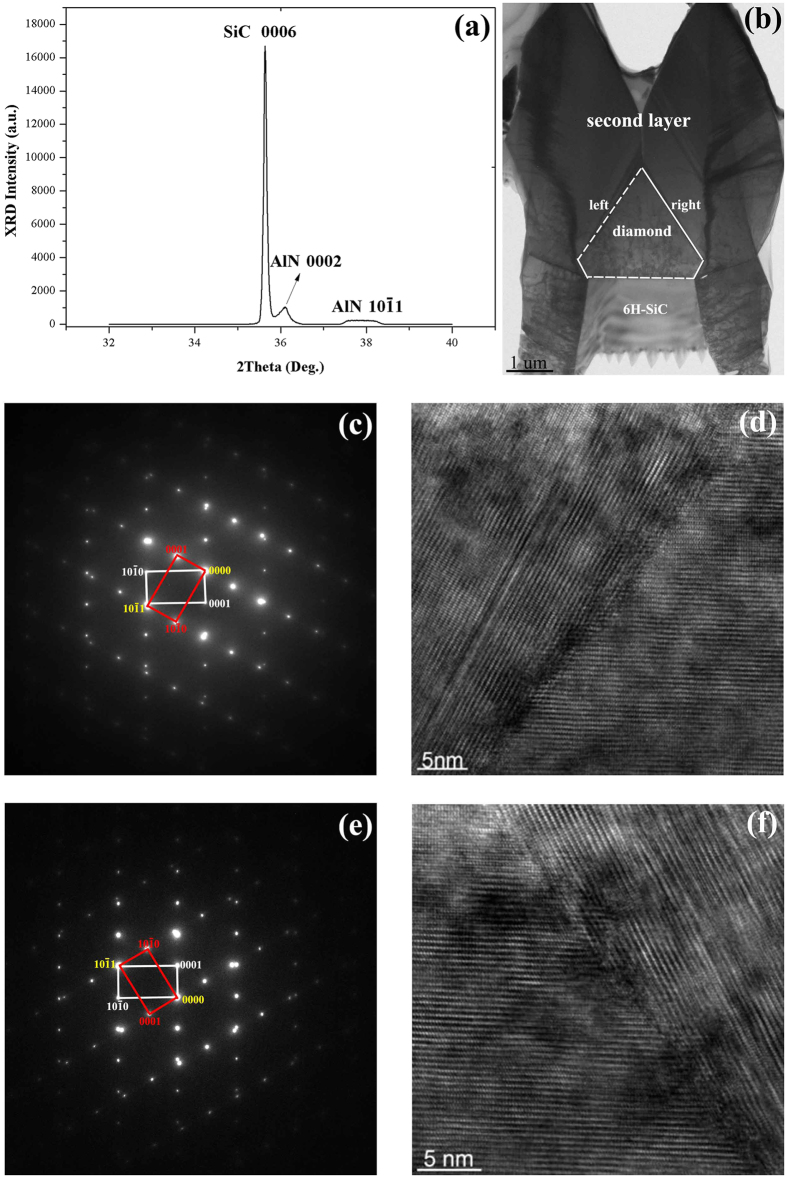
Characterization of AlN film grown on 6H-SiC with 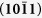 twin boundary. (**a**) XRD 2θ/ω scan of AlN film grown on patterned 6H-SiC by ELOG, which indicates that 0002 and 10
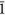
1 diffraction peaks coexist in the film. (**b**) TEM cross sectional image of AlN film. (**c,d**) are SAED pattern and HRTEM image taken from the left interface. (**e,f**) are SAED pattern and HRTEM image taken from the right interface. Both interfaces are (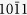) incoherent twin boundaries.

The results propose a new valuable method to prepare semi-polar AlN thick film by twin growth mode. In this method, the key point is to control the growth front and construct the twin structure. It is known that by epitaxial lateral overgrowth (ELOG) technology the growth front of GaN can be easily controlled on trench patterned substrates^25^. On the other hand, the etching of SiC is easier than that of sapphire. Therefore, based on this idea, initial success by ELOG technology on the patterned 6H-SiC substrate has been achieved at growth temperature of 1300 °C, as shown in Fig. 4. X-ray 2θ/ω scan (Fig. 4a) shows that 0002 and 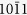 peaks coexist in the AlN film, which proves that the epilayer really contains (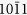)-semi-polar AlN. TEM measurements were performed to study the mechanism of this semi-polar film. As marked in Fig. 4b, this AlN film contains two layers with different crystal orientations. An inverted diamond-like layer is grown from the terraces of the substrate and the second layer is grown from the sides of the diamond-like structure. SAED patterns show that growth directions of the diamond-like layer and the second layer are along [0001] and [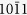], respectively. Figure 4c,e are SAED patterns which taken from the interfaces between the diamond-like structure and the second layers. Since the diffraction spots satisfy the mirror symmetrical relationship while the two-dimensional crystal lattices in the HRTEM images (Fig. 4d,f) do not keep symmetrical, both boundaries are determined as (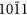) incoherent twin boundaries. Thus, we have artificially controlled the growth of (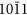) twin structures to realize semi-polar growth in wurtzite AlN. It is believed that (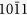) facets on surface are essential to induce the nucleation of (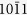) twin structures further.

**Figure 5:**
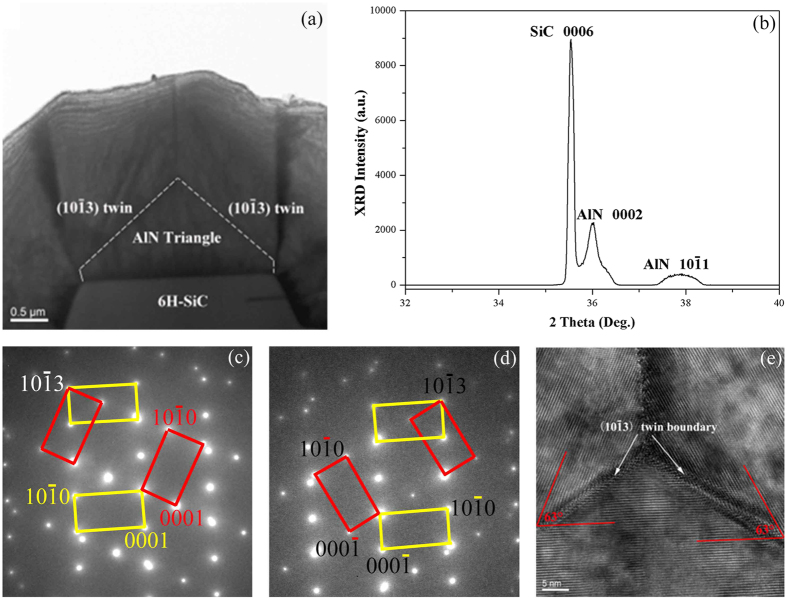
Characterization of AlN film grown on 6H-SiC with (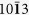) twin boundary. (**a**) TEM cross sectional image of AlN film. (**b**) XRD 2θ/ω scan of AlN film indicates that 0002 and 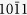 peaks coexist in the film. (**c,d**) are SAED patterns correspond to the left interface and the right interface in (**b**), respectively, which indicate both interfaces are (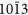) twin boundaries preliminarily. (**e**) HRTEM image taken from the interfaces between triangle and the column, which demonstrates that the two interfaces are (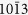) coherent twin boundaries further.

Due to the high bond energy of Al and N, the growth temperatures of high-quality AlN are generally higher than 1400 °C^26^,^27^. For this reason, using the above method, the semi-polar AlN film on trench patterned SiC substrate on elevated temperature of 1400 °C has been grown. Figure 5a shows the cross-sectional TEM image of this AlN film. Obviously a triangle-like layer was grown on terraces of substrate firstly and then the second layer was grown from the first layer. The interface was marked by white dashed line. X-ray 2θ/ω scan (Fig. 5b) indicates that 0002 and 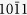 peaks coexist in the AlN film. Attention is paid to the interfaces between the triangle-like layer and the second layer to clarify the connection mode of the two parts. Combining SAED patterns (Fig. 5d,e) and HRTEM image (Fig. 5f), we conclude that the two interfaces are (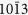) coherent twin boundaries instead of (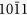) twin boundaries. In other words, [0001]-directed AlN transforms to [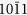]-directed AlN successfully by forming (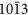) twin structures.

**Figure 6:**
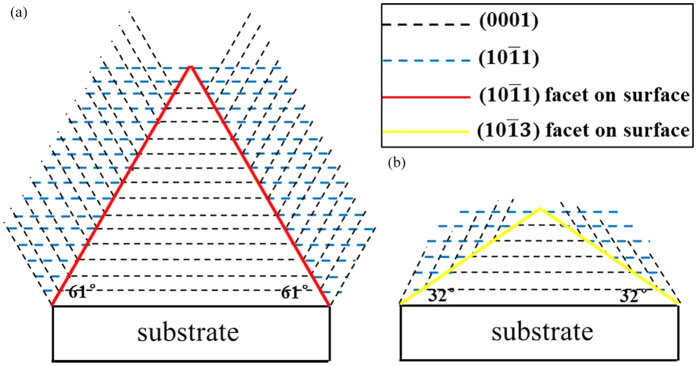
Diagram of semi-polar AlN grown through twin structure. (**a,b**) Schematic of semi-polar AlN growth through (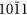) and (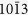) twin growth mode, respectively.

Therefore, [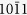]-directed AlN thick films can be grown on (0001) sapphire or 6H-SiC substrates by twin growth mode (both (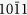) and (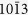) twin structures). The geometrical features of twin structure can change the growth orientation of crystals, which shows schematically in Fig. 6. The black and blue dash lines represent cross section of (0001) and (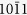) planes, while red and yellow solid lines are those of (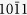) and (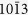) facets on surface, respectively. Considering the interfacial angle between (0001) and (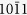) is 61° and the value between (0001) and (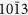) is 32°. If the (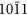) facet on surface is formed firstly (Fig. 6a), it will develop (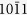) twin structure. If the facet on surface is (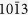) plane (Fig. 6b), then (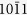) twin structure will be formed. It needs to be pointed out that the real situations may be much more complicated than the simple picture presented here. However, further research work towards this promising method of fabricating other kind of semi-polar AlN thick films may be worthwhile.

In summary, (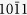) semi-polar AlN were grown on conventional *c-plane* sapphire by HVPE. It was found that twin structures were formed at the interface of (0001)-polar and (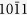) semi-polar layers, which changed the growth direction and resulted in the growth of semi-polar AlN materials. TEM measurements showed that tilt facets occurred on the surface during the growth of the (0001)-polar layer, which may be crucial for the following twin growth mode. Based on the idea, (0001) polar AlN with different tilt facets on the surface were grown on trench-patterned *c-plane* 6H-SiC by ELOG technique. Then (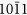) semi-polar AlN was successfully fabricated on the polar layer by forming (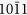) or (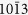) twin structures, which depended on the tilt angle of facets on the surface. This work presented a method for growing semi-polar AlN on conventional *c-plane* sapphire or 6H-SiC. Further research work towards this promising fabrication method of semipolar or nonpolar AlN may be worthwhile.

## Methods

Preparation of AlN on *c-plane* sapphire by HVPE: AlN growth was carried out on home-made horizontal HVPE. The growth system equipped with cold wall quartz tube reactor used induction heating method. (0001)-Sapphire (*c-plane* sapphire) was used as substrate for the AlN growth. HCl and ammonia were used as input active gases. Before AlN deposition, HCl flowed over Al source to form gaseous aluminum chlorides at 550 °C. The mixture of H_2_ and N_2_ (mixed ratio of 1:1) were used as carrier gas. Firstly, the sapphire was cleaned in H_2_ at 1100 °C. Then the temperature was decreased to 800 °C for the 200 nm AlN buffer growth. Finally, thick AlN layer was grown at 1300 °C under the pressure of 40Torr.

Preparation of trench-patterned 6H-SiC substrates: The standard photolithography was used to prepare SiO_2_ stripes separated by windows on SiC substrates. The strips were parallel to the direction of <
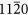
> of SiC. Then the substrates were etched with inductively coupled plasma etch system (ICP 180) to remove the SiC in the windows and to form about 2 μm deep trenches. Finally, SiO_2_ stripes were moved in hydrofluoric acid (HF) and the patterned 6H-SiC substrates were formed with about 2.2 μm terrace separated by 3.8 μm trenches.

Preparation of AlN film on patterned 6H-SiC by epitaxial lateral overgrowth (ELOG): Prior to growth, the patterned SiC substrates were cleaned firstly in H_2_SO_4_ and H_2_O_2_ mixture with volume ratio of 3:1 for 5 minutes at 80 °C. After cleaned in deionized water, the substrates were immersed in hydrochloric acid (HCl), aquaregia and HF in sequence for 10, 10 and 5 minutes, respectively. Subsequently the patterned substrates were loaded in HVPE system for AlN growth. Two growth steps with different V/III ratios of 10 and 5 were selected for one run to get tilt planes of AlN sidewall grown on the terraces and for the following growth, respectively. Two samples were grown at 1300 and 1400 °C, respectively.

TEM Analysis of AlN films: The TEM specimen was prepared by a standard mechanical grinding and ion milling method with a final 3 keV cleaning at 4° in the Fischione Ion Mill in order tominimize the artificial defects introduced by the specimen preparation. The characterization of the cross sectional morphology was performed by TEM on Tecnai G^2^ F20 S-Twin 200KV.

XRDand SEM Analysis of AlN films: The characterization of AlN template was measured by x-ray diffraction (XRD) on a Bruker D8 Discover. The cross sectional morphology of the AlN film was characterized by scanning electron microcopy (SEM) on a Quant 400.

## Additional Information

**How to cite this article**: Liu, T. *et al.* Nucleation and growth of (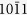) semi-polar AlN on (0001) AlN by Hydride Vapor Phase Epitaxy. *Sci. Rep.* **6**, 26040; doi: 10.1038/srep26040 (2016).

## Supplementary Material

Supplementary Information
