# Supplementary material for: Nucleation and growth of (10¯11) semi-polar AlN on (0001) AlN by Hydride Vapor Phase Epitaxy
Source: Sci Rep. 2016 May 17;6:26040. doi: 10.1038/srep26040 (PMC4877591; doi:10.1038/srep26040)
Supplement: Supplementary Information [file srep26040-s1.pdf]

Nucleation and growth of $(10\bar{1}1)$ semi-polar AlN on (0001) AlN by Hydride Vapor Phase Epitaxy

Ting Liu[1], Jicai Zhang[1,2] *, Xujun Su[1], Jun Huang[1], Jianfeng Wang[1,2], & Ke Xu[1,2]*

[1]Platform for Characterization and Test, Suzhou Institute of Nano-tech and Nano-bionics, CAS, Suzhou, 215123, China.

[2] Suzhou Nanowin Science and Technology Co., Lt/d, Suzhou, 215123, China.

*jczhang2010@sinano.ac.cn, kxu2006@sinano.ac.cn

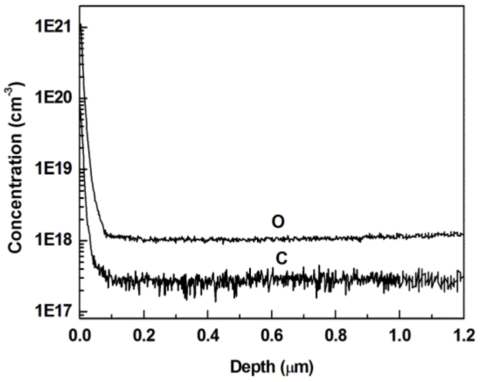

Supplementary Fig. S1 The result of SIMS shows the concentrations of oxygen and carbon are around 3×10^{17} and 1×10^{18} cm^{-3}, respectively.

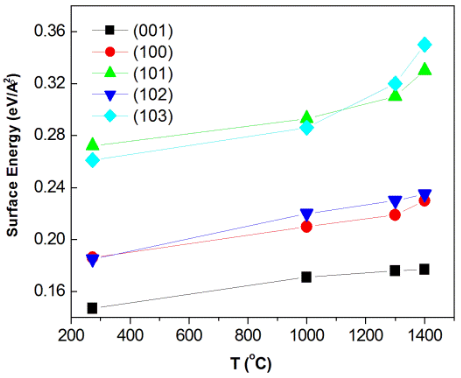

Supplementary Fig. S2 The surface energy of a certain plane as a function of temperature.
